# Regeneration of T cells from human-induced pluripotent stem cells for CAR-T cell medicated immunotherapy

**DOI:** 10.3389/fbioe.2023.1159507

**Published:** 2023-05-18

**Authors:** Yanyan Chen, Pufeng Huang, Mengda Niu, Chuanhuizi Tian, Tingting Zhang, Zhiping Peng

**Affiliations:** Department of Radiation Medicine, School of Basic Medicine, Chongqing Medical University, Chongqing, China

**Keywords:** immunotherapy, cell differentiation, human induced pluripotent stem cells, lymphomagenesis, hiPS-CAR-T cells

## Abstract

**Background:** Chimeric antigen receptor (CAR) T cell treatment involves *in vitro* production of T cells from patient blood with synthetic receptors specific to a cancer antigen. They circumvent the major histocompatibility complex to recognize the tumor antigen, reducing hematologic malignancy remission rates by 80%. Considering the efficacy of CAR-T treatment, the present work aimed at generating functional clusters of differentiation (CD)8 + T cells from human induced pluripotent stem cells (hiPSC) and to generate hiPS-CAR-T cells with high antigen-specific cytotoxicity.

**Methods:** The Alkaline phosphatase assay and MycoEasy rapid *mycoplasma* detection kit was implemented for detection of hiPSCs and *mycoplasma*, respectively. The CD34^+^ HSPCs were harvested in AggreWellTM 400 using a 37-micron reversible strainer. Likewise, the lymphoid progenitor and CD4^+^CD8^+^ DP T cells were also harvested. The Cell Counting Kit-8 (CCK-8) assay was used to mark cytotoxicity and ELISA was used to detect IFN-γ secretion. Further, flow cytometry and transwell chambers were used to assess cell cycle, and migration and invasion. Finally, the *in vivo* antitumor effects of the CAR-T cells were evaluated using experimental animals (mice).

**Results:** Results revealed that a serum-free, feeder layer-free differentiation system significantly yielded hiPSC-based T cell immunotherapy with interleukin-2, interleukin-15, and activators at the differentiation stage to promote the maturation of these cells into human induced pluripotent stem (hiPS)-T cells. The infection of hiPSCs with the CD19 CAR lentivirus resulted in the production of the hiPSC-CAR-T cells. We validated the function of hiPS-CAR-T cells *in vivo* and *in vitro* experimentation which revealed no significant differences in cell morphology and function between hiPSC-derived hiPS-CAR-T cells and peripheral blood-derived CAR-T cells.

**Conclusion:** This study developed a culture method that is efficient and clinically useful to make functional CD8^+^ T cells from hiPSC and to get hiPS-CAR-T cells with high antigen-specific cytotoxicity that are not very different from CAR T cells found in peripheral blood. As a result, our findings may open the way for the clinical use of hiPSC to create functional CD8^+^ T and hiPS-CAR-T cells cells for use in cell-based cancer therapy.

## 1 Introduction

Numerous cancer immunotherapies have been developed (Cancer vaccines, T-cell therapy, Oncolytic virus therapy, etc.), and T cells derived from human hematopoietic stem cells/progenitor cells differentiating from the thymus are extensively used in several treatments (Esfahani et al., 2020). T cell progenitors are expanded under the stimulation of interleukin 7 (IL-7) and delta-like 4 (DLL4), prompting differentiation into T cells once the cells migrate to the thymus (Hozumi et al., 2008). CAR-T immunotherapy is a cancer immunotherapy technology that uses T cells obtained from patients to attack specific receptors on cancer cells.

T cells can be used to treat various diseases, which is confirmed by a large number of basic and clinical research studies. One potential method for expanding the use of this therapy is to develop a ready-made source of T cells. Presently, the sources of such T cells include healthy donors’ peripheral blood and those generated from pluripotent stem cells (PSCs). Currently, it has been reported that hiPSCs can differentiate into multiple cell lines *in vitro*. These include cardiomyocytes (Austin et al., 2019; Podgurskaya et al., 2019), neuronal cells (Mertens et al., 2016), hepatocytes (Takebe et al., 2013)^,^ and insulin-secreting cells (Li et al., 2020), to establish disease models, as well as for drug screening (Kimbrel and Lanza, 2015). As mentioned above, the generation of T cells encompasses a series of differentiation with multiple critical intermediates, which is usually achieved via reproducing signal transduction and hematopoietic stem and progenitor cells (HSPCs) differentiation. HiPSCs are first induced to differentiate into embryoid bodies (EBs) and then HSPCs. Under the DL ligand-mediated signal, generated HSPCs differentiate sequentially into a lymphoid progenitor, T cell progenitor, and T lineage cells, which can be observed in the human thymus (Spits, 2002; Awong et al., 2009).

In the immune system, tumor-targeting CAR-T cells can be cultured via modified CAR composition, e.g., transfection of CAR lentivirus (Barthélémy et al., 2015; Zhang et al., 2016; Martinez and Moon, 2019). As for most of the procedures that induce hiPSCs to differentiate into T cells, a protocol using serum and feeder cells (OP9-DL1 or C3H10T1/2) is being used currently (Vizcardo et al., 2013; Minagawa et al., 2018; Nishimura and Nakauchi, 2019). Each feeder cell layer requires a different serum-containing basal medium to maintain a viable culture. These are co-cultured with differentiating hiPSCs, making reproducibility challenging. At the same time, this method has an increased risk of viral and bacterial contamination. If these cells are used in clinical therapy, they must remain contagion free. This makes the direct clinical use of these cells expensive and nearly impossible in some cases, especially if the culture fails. Thus, it is crucial to use a serum-free and feeder-free (Ff) differentiation system for hiPSCs-based T cell immunotherapy during the differentiation stage.

**FIGURE 1:**
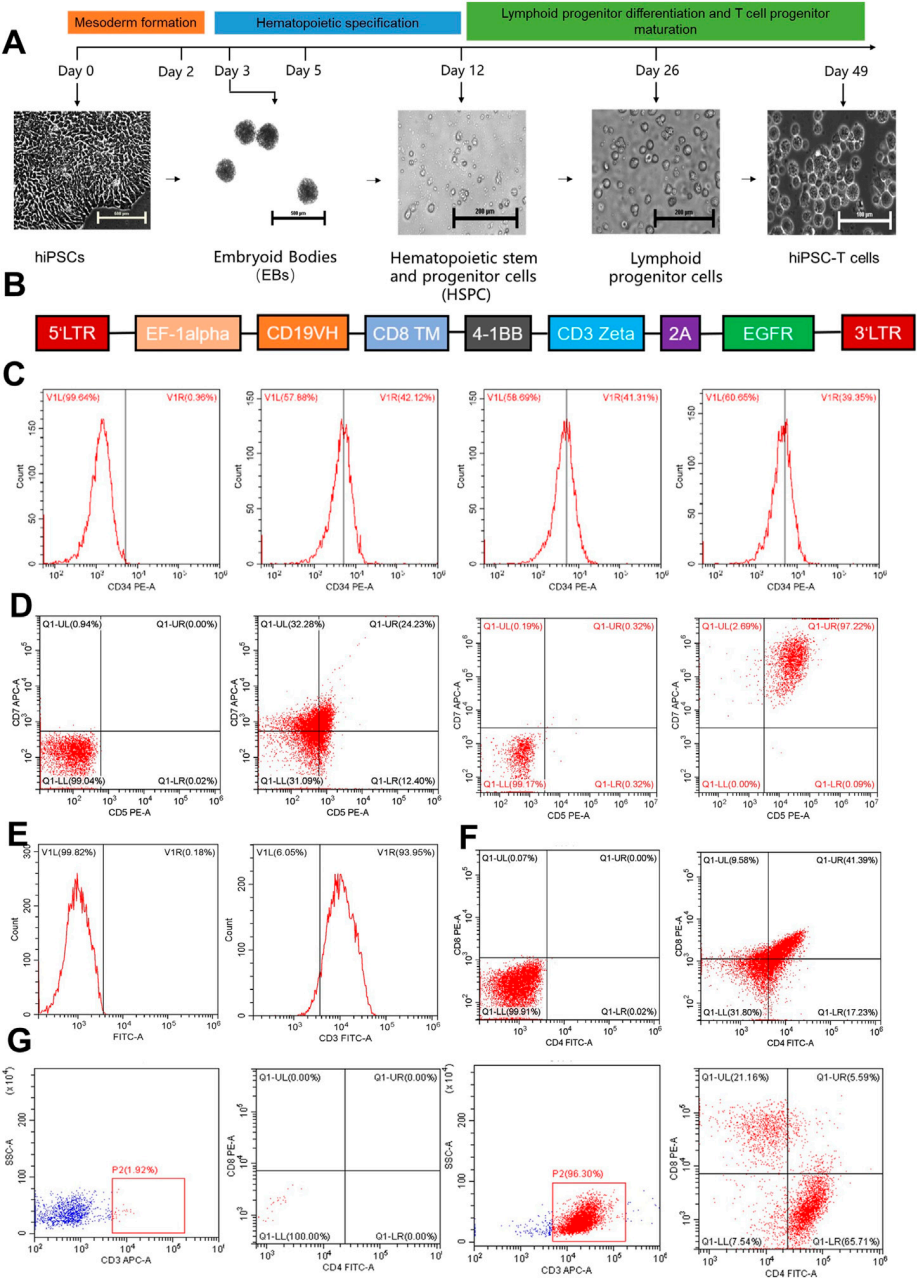
The figure represents the differentiation of hiPCS into T lymphocytes and the preparation of hiPS-CAR-T cells. **(A)** *In vitro* T lymphocyte differentiation protocol: directed differentiation of hiPSCs into hiPS-T cells includes three steps. 1) Mesoderm formation (day 1–3), 2) hematopoietic specification (day 4–12), and 3) lymphoid progenitor differentiation and T cell progenitor maturation (day 13–49). **(B)** The sequence of the CD19 CAR lentiviral vector. **(C)** On day 12, the differentiated HSPCs were analyzed by flow cytometry and revealed relatively high expression of CD34^+^. **(D)** On day 26 of differentiation, CD7^+^CD5^+^ lymphoid progenitor cells were detected without or with IL-15. **(E)** On day 42, the CD3^+^ cells accounted for most of the differentiated cultured cells analyzed by flow cytometry. **(F)** Simultaneously, CD4^+^CD8^+^ DP expression was also found in the differentiated T cell lineage. **(G)** After 7 days of exposure to a differentiation medium containing IL-2, IL-15, and T cell activator, Single-positive T cells (CD8^+^)were harvested and confirmed by flow cytometry.

The objective of the current study was to produce operational clusters of differentiation (CD)8 + T cells utilizing hiPSC and to generate hiPS-CAR-T cells that exhibit elevated antigen-specific cytotoxicity. We used an Ff differentiation technique for hiPSCs-based T cell immunotherapy to facilitate cell maturation into CD8^+^ hiPS-T cells (Figure 1A). Further, the study assessed the cytotoxicity of the expanded hiPS-CAR-T cells using *in vitro* and *in vivo* experiments to find out whether hiPSCs can be a ready-made T cell source product. Results demonstrated that this approach produced high quantities of hiPSC CAR-T cells and suppressed the growth of Ranji or A549 cancer cells in culture and *in vivo*.

## 2 Materials and methods

### 2.1 Chemicals, reagents, and cell lines

Experimental hiPSCs were provided by CellapyBio (Beijing, China), with 1 × 10^6^ hiPSCs in each vial. Cells were thawed in a constant temperature water bath at 37°C before each experiment. If not stated otherwise, all of the chemicals and reagents used in this work were purchased from Sigma-Aldrich in St. Louis, MO, United States of America.

### 2.2 Animals

BALB/c nude mice (n = 15) were purchased from Beijing Vital River Laboratory Animal Technology Co., Ltd. The mice were housed under specific pathogen-free conditions, at 25°C, 60%–80% humidity, 12-h light/dark cycles, and free access to food and water. Food intake and body weight of the experimental animals were continuously recorded (Table 1; Table 2, respectively). The care of the experimental animals involved in this study conformed to the various requirements of the principles of medical ethics and the Declaration of Helsinki. The study design had a scientific basis, and the species, grade, number, and specifications of the animals selected were appropriate. The animals were treated humanely during the experiments, given anesthesia and analgesia, and other therapeutic treatments, and the animals were disposed of harmlessly after death without causing harm to the environment. The study was approved by the Medical Research Ethics Committee of Chongqing Medical University.

**TABLE 1:** Food intake of experimental animals expressed in g/day from day 0–80.

		PBS			T cells			CAR-T cells			hiPS-T cells			hiPS-CAR-T cells		
		1	2	3	1	2	3	1	2	3	1	2	3	1	2	3
Day 0 (no tumor)	Prefeedin	36.7	36.5	37.8	37.5	39.2	39.6	42.6	42.9	38.6	38.3	30.9	39.1	30.5	39.1	36.1
	After feedin	31.8	31.2	33.1	31.9	34.8	34.7	37.5	37.9	33.7	33.7	25.7	33.8	25.5	34.2	30.9
	Feed intake	5.9	6.3	5.7	6.6	5.4	5.9	6.1	6	5.9	5.6	6.2	6.3	6	5.9	6.2
Day 14 (24 h post tumor)	Prefeedin	31.9	33.7	32.3	31.6	31.8	33	31	32	30.2	31.9	32.7	35.3	34.8	35.3	35.8
	After feedin	28	29.1	28.1	26.5	27.7	28.6	26	26.2	25.2	27.7	28	30.6	29	29.6	29.7
	Feed intake	3.9	4.6	4.2	5.1	4.1	4.4	5	5.8	5	4.2	4.7	4.7	5.8	5.7	6.1
Day 28	Prefeedin	33.9	31.3	40.1	30.2	30.1	29.9	21.9	23.6	26.1	36.8	28.5	26.8	36.5	35.9	33.9
	After feedin	32.9	28.2	36.5	25.6	26.2	25.8	17.3	18.9	22	33	24.6	23	30.9	30.2	28
	Feed intake	1.0	3.1	3.6	4.6	3.9	4.1	4.6	4.7	4.1	3.8	3.9	3.8	5.6	5.7	5.9
Day 42	Prefeedin	—	25.9	30.5	35.9	35.3	34.7	36.8	38.5	37.5	21.9	29	23.6	29.6	22.6	31.1
	After feedin		24	27.6	33	33.1	31.1	33.4	33.7	33.2	18.2	25.2	19.8	23.1	16.6	25
	Feed intake		1.9	2.9	2.9	2.2	3.6	3.4	4.8	4.3	3.7	3.8	3.8	6.5	6	6.1
Day 56	Prefeedin	—	—	20.6	31	36.8	30.4	29.9	26.9	30.3	31.3	32	36	30.6	30.7	29.5
	After feedin			18.8	29.7	35.9	26.8	28.3	21.7	25.4	27.3	27.9	32	24.7	24.9	23.6
	Feed intake			1.8	1.3	0.9	3.6	1.6	5.2	4.9	4.0	4.1	4	5.9	5.8	5.9
Day 80	Prefeedin	—	—	—	—	—	35.1	—	34.1	36.9	30.2	33	36.1	22.6	30.2	33.9
	After feedin						33.3		29.2	32.7	28.1	29.1	33.5	16.7	24.1	28.6
	Feed intake						1.8		4.9	4.2	2.1	3.9	2.9	5.9	6.1	5.3

**TABLE 2:** Continuous weight (g) assessment of experimental animals from day 14–80.

	PBS			T cells			CAR-T cells			hiPS-T cells			hiPS-CAR-T cells		
	1	2	3	1	2	3	1	2	3	1	2	3	1	2	3
Day 14	18.9	19.7	19.3	20.1	18.1	17.9	19.8	18.8	19.2	19.1	19.3	19.3	19.3	19.2	19.2
Day 28	15.6	20.1	19.7	19.7	17.5	18.2	20.1	18.5	19.5	19.3	19.1	18.9	19	19.1	19.5
Day 42	—	17.6	18.1	18.8	15.3	18.2	19.7	18.3	18.7	18.7	19.1	18.7	19.7	19.2	19
Day 56	—	—	17.4	17.4	14.4	17.9	17.9	18.7	19	19	19.3	18.9	19	19	19.3
Day 80	—	—	—	—	—	17.3	—	18.7	19.1	17.9	19.2	18.1	19.3	19.1	19.1

### 2.3 Alkaline phosphatase (AP) detection of hiPSC

AP Staining Kit (Sigma, SCR004), 4% paraformaldehyde universal tissue fixation solution (Biosharp, BL539A), and sterile PBS (BOSTER, PYG0021) were used. The specific experimental method was carried out according to the kit operation manual (when the fixed iPS cells were stained, the undifferentiated cells were red or purple, while the differentiated cells were colorless). (Ji et al., 2022).

### 2.4 *Mycoplasma* detection

*Mycoplasma* agarose gel electrophoresis: MycoEasy rapid *mycoplasma* detection kit (CA6101024); TAE (50X) (purchased from Biyuntian, Art. No. ST716); Agarose (BIOFROXX, Art. 1110); DNA ladder (0.1–10 kB, 21 bands, with Beyc C) purchased from Biyuntian Art D0109; GoodView™ Nucleic Acid Dye (purchased from Cypax HGV-II); the Collect the cell culture supernatant to be tested, and the subsequent experimental operation was carried out according to the operation manual of the *mycoplasma* detection kit. Note: The positive standard for *mycoplasma* is about 280 BP, and the primer dimer was about 80 BP. (Wu et al., 2022).

### 2.5 Generation of single-cell suspension

Culturing of HiPSCs of 5 × 10^5^ cells/well concentration with normal proliferation rate and shape was accomplished. Then, a single hiPSC suspension was prepared by washing the cultured hiPSCs with sterile Dulbecco’s phosphate buffer saline (D-PBS), adding 5 mL/well of Accutase™. After gently removing the remaining attached cells, the fraction of cells was transferred into a 15 mL centrifuge tube. Finally, the culture plate was washed with 10 mL of Dulbecco’s Modified Eagle Medium (DMEM)/F12 containing 15 mM N-2-hydroxyethylpiperazine-N′-2-ethanesulfonic acid (HEPES) and added to the centrifuge tube, centrifuged at 260 g for 6 min, and resuspended in 2.5 mL of EB supplement (STEMdiff™ Hematopoietic-EB Basal Medium, EB Supplement A, Y-27632) to get 3.5×10^6^ cells/well. The cells were cultured in an AggreWell™ 400 6-well plate at a density of 3.5×10^6^ cells/well and centrifuged at 100 g for 3 min. The plated cells were incubated at 37°C with 5% CO_2_ for 2 days. On day 3, STEMdiffTM hematopoietic-EB basal medium and EB supplemented medium B was used for half-liquid replacement and incubated for 2 days.

### 2.6 Harvest of CD34^+^ HSPCs

A 37-micron reversible strainer was placed in each AggreWell™ 400 (Sigma, St. Louis, United States) well from which EBs were harvested, the EB suspension was transferred to a filter, and the well’s surface was rinsed with DMEM/F12 (Sigma, St. Louis, United States) to collect any remaining EBs. Flip the 37-micron mesh and place it on a clean centrifuge tube. Embryoid bodies collected on the filter were resuspended in a medium and incubated for 2 days. Half-chance the medium with 2.5 mL/well of EB Medium B every 2–3 days. Collagenase II solution was prepared (2,500 U/mL, #12,604-013; Thermo Fisher, United States). The EBs were resuspended in collagenase II solution. Then, 3 mL of TrypLETMExpress (Thermo Fisher Scientific, United States) was added and mixed, and incubation was continued. After that, the cells were centrifuged at 200–300 g for 3–5 min. The cells were resuspended in RoboSepTM buffer (Stemcell, #20104) and incubated according to the EasySepTM human CD34 positive selection kit II (Stemcell, #17856) technical manual for CD34 positive cell sorting and purification.

### 2.7 Harvest of lymphoid progenitor cells

First, 500 µL of coating material (coating material ×100 + D-PBS without Ca^2+^ and Mg^2+^) was added to each well and incubated at 25°C for 2 h. The obtained HSPCs were resuspended with a lymphoid progenitor expansion medium (StemSpan™ SFEM Ⅱ, StemSpan™ Lymphoid Progenitor Expansion Supplement) using the following steps: Firstly, 90% of the culture medium was resuspended with thawed StemSpan™ SFEM II at 4°C overnight, and 10% of the culture medium was resuspended with thawed StemSpan™ Lymphoid Progenitor Expansion Supplement ×10 at room temperature and cultured. Half of the medium was replaced with a lymphoid progenitor expansion medium every 3–4 days, and after 14 days following pipetting and centrifugation, it was resuspended with T cell progenitor maturation medium (StemSpan™ SFEM Ⅱ, StemSpan™ T Cell Progenitor Maturation Supplement) and cultured in lymphoid differentiation material-coated plates at a density of 5 × 10^5^ cells/well for differentiation using manufacturer protocol. The cells were transferred to a 15-mL centrifuge tube. These cells included the lymphoid progenitor cells as well.

### 2.8 Harvest of CD4^+^CD8^+^ DP T cells

During culture, half of the medium was gently replaced with 500 μL/well of T cell progenitor maturation medium every 3–4 days. After 14 days, the cells were gently blown to ensure that all cells were suspended in culture before being transferred to a 50-mL tube to harvest CD4^+^CD8^+^ DP T cells as follows: To begin, the expression of CD4 and CD8 was detected based on the morphological appearance of cells under the microscope, as well as the expression of specific marker CD3 on the cell surface by flow cytometry. The cells were gently pipetted up and down. This cell suspension included CD4^+^CD8^+^ DP T cells as well. In the case of cells with CD3 positivity >90%, cells were not isolated and purified, which was followed by the next step of the CD8^+^ SP T cell maturation culture protocol.

### 2.9 Harvest of CD8^+^ SP T cells and hiPS-CAR-T cells

The harvested DP T lymphocytes were transferred to lymphoid differentiation material-coated plates, and 10 ng/mL IL-2, 10 ng/mL IL-15, and 6.25 µL human CD3/CD28/CD2 T cell activator were added to each well for 7–14 days for maturation and differentiation of CD8^+^ T cells (Minagawa et al., 2018; Hu et al., 2020; Ji et al., 2022; Wu et al., 2022; Jian et al., 2023). Except for mature T cells, most immature lymphocytes, including CD4^+^CD8^+^ DP T cells, died as a result of T cell receptor (TCR) rearrangement. CD3^+^ T cells were sorted out using the CD3 Positive Selection Kit II (stemcell#17851), followed by CD4^−^CD8^+^ T cells (hiPS-T) using the CD4 Positive Selection Kit (stemcell, #17852) and CD8 Positive Selection Kit (stem cell, #17853). From the CD3^+^ T cells obtained above, CD4^−^CD8^+^ T cells (hiPS-T) were isolated, purified, and set aside. The harvested hiPS-T cells were infected with CD19 CAR lentivirus (5 μg/mL polybrene + CAR lentivirus (MOI = 7.5)) (Figure 1B) for 24 h to obtain hiPS-CAR-T cells.

### 2.10 Killing and cytokine release abilities of hiPS-CAR-T

Raji cells expressing CD19 antigen and A549 cells not expressing CD19 antigen were used as target cells (cell count was 1 × 10^5^). The hiPS-T cells and T cells isolated from peripheral blood were infected with the CD19 CAR lentivirus to prepare hiPS-CAR-T cells and CAR-T cells, respectively. These four groups of cells served as effector cells for the subsequent experiments. The effector and target cells were mixed and cultured according to different effector-target ratios (0.5∶1, 1∶1, 2∶1, 5∶1, 10∶1), and morphological changes of cells after co-culture were observed under the microscope. The collection of supernatant of cell co-culture was done, and the cytotoxicity was detected by CCK-8 kit (#E-CK-A362; Elabscience, Texas, United States), the absorbance OD value at 450 nm was detected by a microplate tester, and the cell inhibition rate was calculated. ELISA was used to detect IFN-γ secretion in each group at the same time.

$$Inhibition\;Ration=\left(ODcontrol-ODsample\right)÷\left(ODcontrol-ODblank\right)\times 100\%$$

### 2.11 Flow cytometry

According to the manufacturer protocol, cells and CD19 CAR lentivirus infected cells were washed and stained with PE anti-human CD5 (BioLegend), APC anti-human CD7 (BioLegend), PE anti-human CD8 (BioLegend), FITC and APC anti-human CD3 (BioLegend), FITC anti-human CD4 (BioLegend), PE anti-human CD34 (Elabscience), and/or APC and FITC anti-human EGFR (BioLegend) antibodies at 4°C in the dark for 30 min. They were analyzed using a BD Influx flow cytometer (Beckman CytoFLEX, California, United States). Maintaining the standard manufacturer protocol, the cell cycle in Raji and A549 cells was also analyzed using flow cytometry. (Jian et al., 2023).

### 2.12 Transwell chamber experiment

The effector cells (T, CAR-T, hiPS-T, hiPS-CAR-T) were treated with E/T = 10/1 for 24 h and the tumor cells in each group were collected. The treated CD19-negative A549 cells were inoculated into a 12-micron membrane (transwell 12-well plate) with 100 μL of cell suspension per well (5×10^5^ cells/mL). CD19-positive Raji cells were also inoculated into another 12-well transwell plate in the same way. The tumor cells were inoculated in the upper compartment, and the medium did not contain fetal bovine serum. The lower chamber contained a high-nutrient medium with 30% fetal bovine serum. After Transwell plates were cultured at 37°C and 5% CO_2_ for 24 h, the volume of tumor cells in the lower compartment was observed. Tumor cells in the lower chamber were treated with a crystal violet dye solution and observed under a microscope. The A549 adherent cells in the lower chamber were fixed with 4% paraformaldehyde for minutes before being washed three times with PBS for 5 min each. The cells were covered with crystal violet staining solution, stained at room temperature for 10 min, and then observed under a microscope after full washing with PBS. Raji cells suspended in the lower chamber were mixed with a 10:1 crystal violet staining solution for 10 min before being observed under the microscope after direct drops.

OD measurements: The tumor cells in the lower chamber stained with crystal violet were decolorized with 33% acetic acid, and after full oscillatory elution, the eluent was moved to the transparent plate of a 96-well plate, and the absorbance OD value of 570 nm was measured with an enzyme marker (OD value was positively correlated with the number of cells migrated to the lower chamber), and the plots were drawn.

### 2.13 Optical *in vivo* imaging

The tumor model is Matrigel: Raji-Luc + tumor cell suspension = 1:1 mixed and then injected into the middle and posterior subcutaneous of the axilla of nude mice, and tumors can be formed in 14 days. In 5 groups of 3, immune cells were injected into tumor-bearing mice through the tail vein. Tumor-bearing mice in group A were infused with 100 µL PBS per mouse, tumor-bearing mice in group B were infused with 100 μL T cells per mouse, tumor-bearing mice in group C were infused with 100 μL CAR-T cells per mouse, and tumor-bearing mice in group D were infused with 100 μL hiPS -T cells/mouse, tumor-bearing mice in group E were infused with 100 μL hiPS-CAR-T cells/mouse (each 100 μL cell suspension contained 1 × 10^7^ cells).

### 2.14 Statistical analysis

Data were expressed as mean ± standard deviation, and GraphPad Prism 8 software was used for data image processing and statistical analysis. The One-Way ANOVA was used to compare data among multiple groups. The *p*-values of *p < 0.05 and **p < 0.001 were considered statistically significant. Each group of experiments was repeated at least three times.

## 3 Results

### 3.1 Differentiation of hiPSCs in a serum- and feeder-free culture producing hiPS-CAR-T cells

**FIGURE 2:**
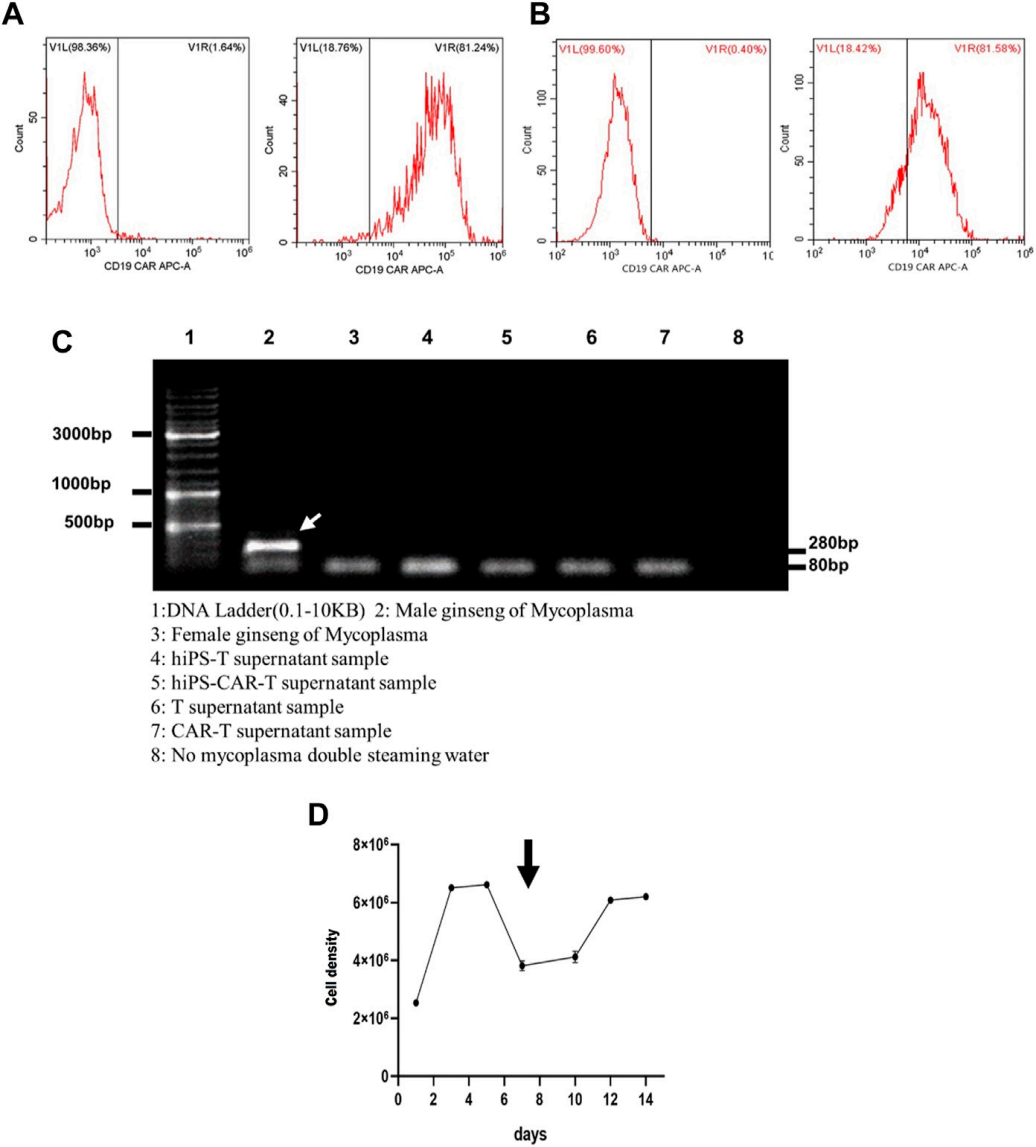
The figure represents lymphocyte culture and the tumor cell killing assay *in vitro*. **(A)** After 24 h of CD19 CAR lentivirus infecting hiPS-T cells, many hiPS-CAR-T cells were harvested. **(B)** The infection efficiency of CAR lentivirus in peripheral blood-derived CAR-T cells was close to that of hiPS-CAR-T cells, as proven by flow cytometry. **(C)** *Mycoplasma* was detected by hiPS-T cells, hiPS-CAR-T cells, peripheral blood-derived T cells, and CAR-T cells. The results showed that there was no *Mycoplasma* pollution. The next step of verification experiments, such as *in vitro* and *in vivo*, can be carried out. 1: DNA ladder (0.1–10 kB). 2: Male ginseng of *Mycoplasma*. 3: Female ginseng of *Mycoplasma*. 4: hiPS-T supernatant sample. 5: hiPS-CAR-T supernatant sample. 6: T supernatant sample. 7: CAR-T supernatant sample. 8: No *mycoplasma*, double-steaming water. **(D)** Activated and expanded hiPS-CAR-T cells after 7 days of culture in 1640 complete medium.

The hiPSC were subjected to an alkaline phosphatase (AP) assay to evaluate lymphocyte differentiation. iPS cells that had not undergone differentiation had very high alkaline phosphatase activity (AP). Positive results for the cell AP labeling indicated that the cells continued to exist in an undifferentiated stem cell state (Supplementary Figure S1A). The hiPSCs that were going to be differentiated as well as the CD19^+^ Raji-luc + cells were tested for the presence of *mycoplasma* as was necessary before the establishment of tumor-bearing animal models. Both types of cells tested negative for *mycoplasma* infection, indicating that they were suitable for use in further hiPSC differentiation and the formation of a tumor-bearing mouse model. The findings demonstrated that the hiPSCs could be differentiated into tumor-bearing animal models. (Supplementary Figure S1B). According to the findings of flow cytometry, CD34^+^ HSPCs were expressed at a rate that was higher than 39 percent (Figure 1C). Using the CD34 Positive Selection Kit, the isolated and purified CD34^+^ HSPCs were further differentiated into lymphoid progenitor cells with an expression rate of CD5^+^CD7^+^>20%. When IL-15 (10 ng/mL) was added during the differentiation of lymphoid progenitor cells, the positive expression rate of CD5CD7 could reach more than 95% (Figure 1D). Finally, the CD3^+^ expression rate of hiPSCs-derived DP-T cells was >90% (Figure 1E), and the CD4^+^CD8^+^ expression rate was >40% (Figure 1F). After differentiation (Figure 1G), the cell suspension was sorted by CD4 Positive Selection Kit (stem cell,# 17852) to remove the CD4^+^ T cells plus the CD8 Positive Selection Kit (stem cell #17853) to collect CD8^+^ T cells, and CD4^−^CD8^+^ T cells were collected. The harvested CD8 SP hiPS-T cells were infected with CD19 CAR lentivirus. The infection efficiency of CAR lentivirus was >80%, as detected by flow cytometry after 24 h (Figure 2A). Therefore, the results obtained from AP staining, *mycoplasma* detection, flow cytometry, and Western blotting, all revealed that the differentiation of the hiPSCs in a feeder and serum-free model yielded the hiPS-CAR-T cells without contamination. These cells were collected and prepared for culture expansion and other studies.

### 3.2 Culture expansion and hiPS-CAR-T cell identification

Both hiPS-T and T cells had an efficiency of CAR lentivirus infection that was below 80% (see Figures 2A, B for further details). *Mycoplasma* testing was performed on differentiated hiPS-T cells, hiPS-CAR-T cells generated by late CD19 CAR lentivirus infection and peripheral blood T cells, and CAR-T cells prepared by infection. All three types of hiPS-T cells and CAR-T cells were negative for the presence of *mycoplasma*. According to the findings, not a single one of the aforementioned four varieties of cells was infected with *mycoplasma* (Figure 2C). For 14 days, the hiPS-CAR-T cells were grown in a medium consisting of 79% RPMI-1640 (#REF C11875500BT, 500 mL; Gibco, United States), 20% FBS (#c0234, Beyotime), 1% penicillin-streptomycin (#15140–122; Gibco, United States), and 500 IU/mL IL-2 (#78036, 50 μg; Stemcell, United States) At this period, the majority of immature lymphocytes perished as a result of TCR rearrangement, whereas mature T cells survived. The differentiated hiPS-CAR-T cells were cultured in media containing activator and IL-2, which expanded 2-3-fold (Figure 2D).

### 3.3 Antigen-specific cytotoxicity by the expanded hiPS-CAR-T cells

**FIGURE 3:**
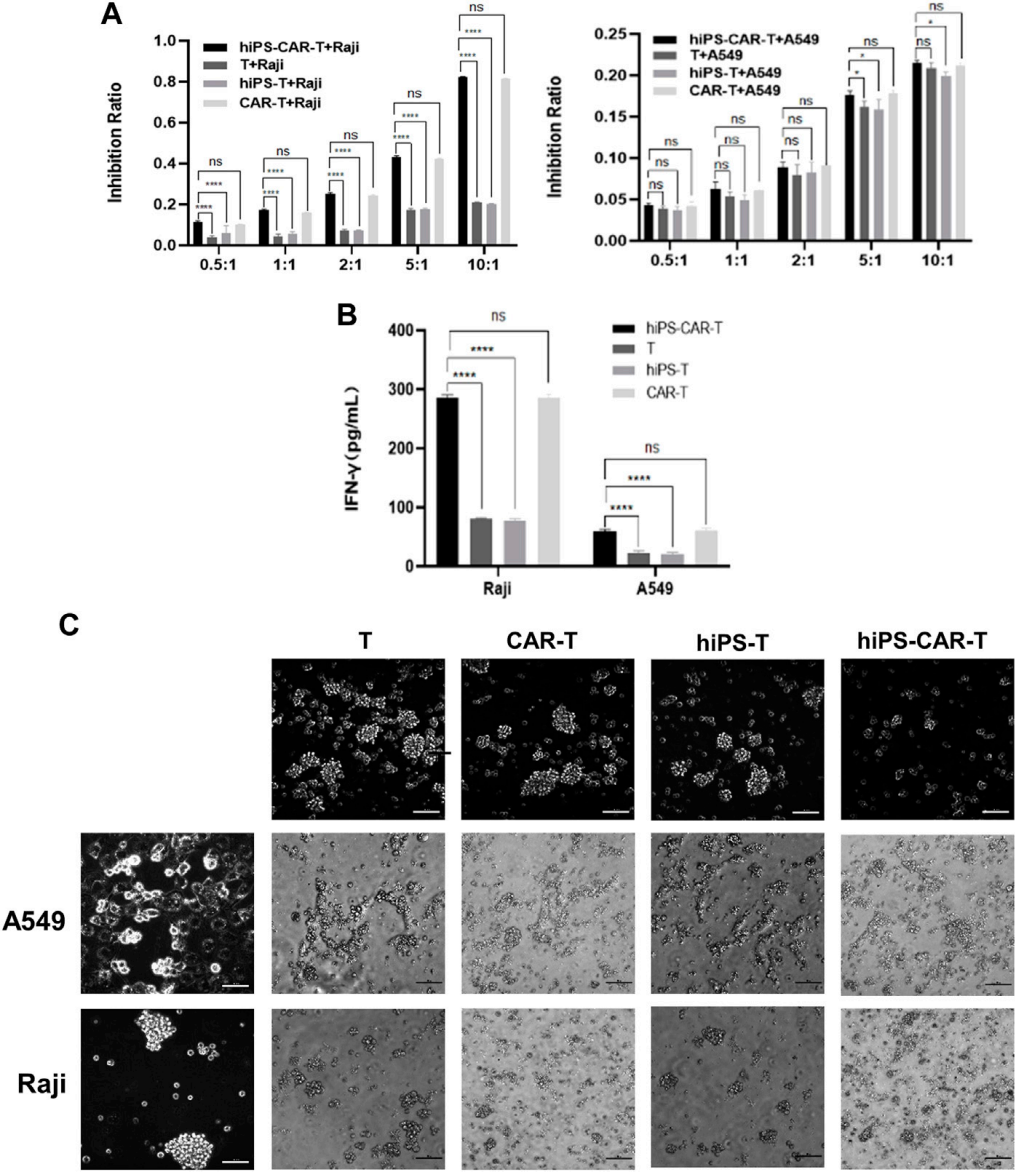
The figure represents antigen-specific cytotoxicity of hiPS-CAR-T cells **(A)** *In vitro* co-culture of hiPS-CAR-T cells, hiPS-T cells, CAR-T cells, and T cells with CD19-expressing Raji cells or A549 cells. The cytotoxicity of hiPS-CAR-T cells was verified by *in vitro* cell killing assay (CCK8). *p < 0.05. ****p < 0.001. NS, no statistically significant differences. **(B)** When the effective target ratio was 10:1, the supernatant of each group was detected by ELISA. The results showed that when the target cells were Raji cells, the IFN-γ release of each group was higher than that of A549 cells. There was no significant difference between hiPS-CAR T cells and CAR T cells, indicating strong cell killing. ****p < 0.001. NS, no statistically significant differences. **(C)** After co-culture with tumor cells for 24 h under the inverted fluorescence microscope, the morphology of tumor cells after hiPS-CAR-T cells or hiPS-T cells co-culture was shown to support tumor cell killing (no significant difference with peripheral blood CAR-T cells and T cells). The change in tumor cells co-cultured with hiPS-CAR-T cells was evident.

**FIGURE 4:**
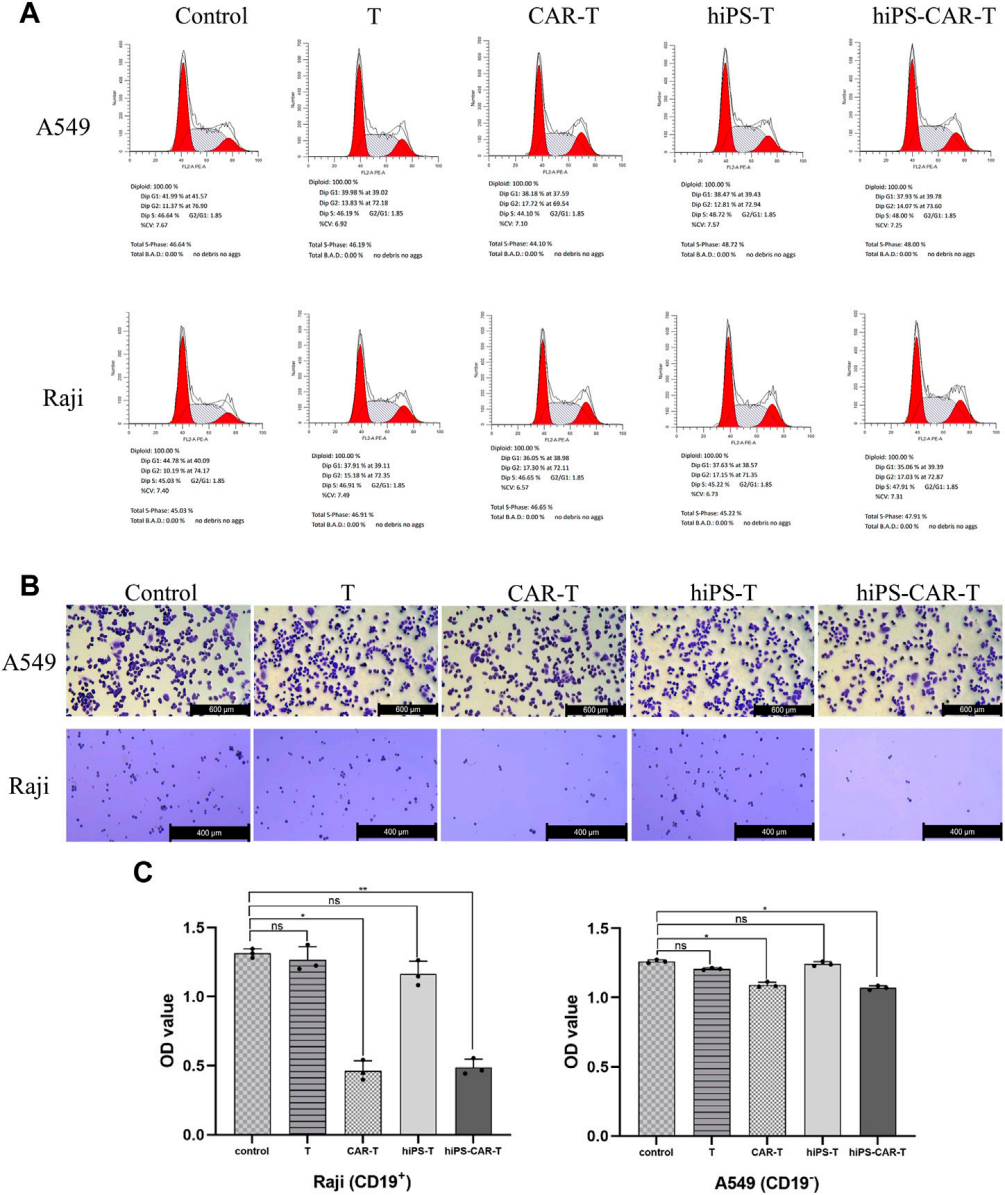
The figure represents transwell chamber experiment for migration and invasion assessment. **(A)** Tumor cells were collected for flow cytometry after 24 h of treatment with tumour cells at an effect-target ratio of 10:1. Results revealing cell cycle blockade while reduction in G1 phase cells. **(B)** It was intuitively discovered that effector cells impede tumor cell death. The experimental group inhibited the development of CD19-positive Raji cells, while hiPS-CAR-T cells inhibited the growth of CD19-positive Raji cells the most effectively. **(C)** Results indicated that T, CAR-T, and hiPS-CAR-T cells inhibited the migration and invasion of CD19-positive Raji cells, with hiPS-CAR-T cells having the largest inhibitory impact. The OD calculations revealed that hiPS-CAR-T cells inhibited tumor cell death more effectively than T cells and the control group.*p < 0.05 and **p < 0.001.

CD19 CAR seeks for and destroys only CD19-positive tumor cells (Raji has CD19^+^ tumor cells, whereas A549s tumor cells are CD19^−^). According to the findings of the CCK8 test, T cells that carried CAR were able to limit Raji cell proliferation at a greater rate than regular T cells working alone. Because CARs are designed to target CD19, the T, CAR-T, hiPS-T, and hiPS-CAR-T cells were co-cultured with CD19^+^ Raji cells and CD19-A549 cells, respectively. According to the findings of the CCK8, there was not a significant difference between the inhibition rates of hiPS-T cells produced from hiPSC and T cells recovered from peripheral blood. There was not a significant difference in the inhibition rate between hiPS-CAR-T which was created by hiPSC differentiation and CAR-T which was made from T cells isolated from peripheral blood. When cells were co-cultured with CD19+Raji cells, the inhibition rate of cells with CAR was much greater than the inhibition rate of cells without CAR. This was seen in the co-culture group. When comparing the killing inhibition of A549 with and without CAR, there was no discernible difference between the two. The killing ability of A549 with CAR may be higher (because CAR improves the targeting and killing ability), and the killing inhibition increases with the increase in the T-cell ratio (when there is a constant number of tumor cells). On the other hand, the killing rate of target cells increases with the increase in the effective target-cell ratio. When the effector-target ratio was set to 10:1 (shown in Figure 3A), the hiPS-CAR T cells were able to attain their highest level of killing efficiency. This was determined by using ELISA to analyze the supernatant of each group. The results showed that when the target cells were Raji, the IFN-γ release of each group was higher than that of the A549 group. In addition, HiPSC-derived hiPS-T cells (IFN-γ concentration was 78.22 ± 1.99 pg/mL) and hiPS-CAR-T cells (IFN-γ concentration was 285.06 ± 4.55 pg/mL) were compared with peripheral blood T cells (IFN-γ concentration was 81.22 ± 0.87 pg/mL), and CAR-T cells (IFN-γ concentration was 286.10 ± 8.03 pg/mL) showed no significant difference in IFN-γ release (*γ*) after co-culture with Raji cells (Figure 3B). The above four effector cells were co-cultured with A549 cells. The release of IFN-γ in each group was consistent with the trend seen in the Raji co-culture group (i.e., no significant differences between T and hiPS-T cells, and no significant differences between CAR-T and hiPS-CAR-T cells), but it was lower than in the Raji group. The four effector cells (T, CAR-T, hiPS-T, and hiPS-CAR-T) were co-cultured with Raji and A549, and the tumor cell morphology supported tumor cell killing when observed under the microscope (Figure 3C). Tumor cells were collected for flow cytometry after being exposed to an effect-target ratio of 10:1 for 24 h. Post-treatment, the cell cycle was found to be blocked and their G1 phase was diminished, which did not support cell growth (Figure 4A). Compared with the control no-treatment group, T, CAR-T, and hiPS-CAR-T cells showed inhibitory effects on CD19-positive Raji cells, and hiPS-CAR-T cells showed the strongest inhibitory effect on the migration and invasion of tumor Raji cells (Figures 4B, C), which was significantly higher than the control no CAR group. There was also an inhibitory effect on CD19-negative A549 tumor cells, but it was not as significant as in the Raji group, most likely because A549 does not express CD19 (our CAR targets CD19) (Figures 4B, C). The experimental results also proved that hiPS-CAR-T cells had a better killing inhibition effect on tumor cells than T cells and no treatment group, and there was no significant difference between stem cell differentiation-derived CAR-T and peripheral blood-derived CAR-T.

### 3.4 *In vivo* inhibition of tumor growth by CAR-T

**FIGURE 5:**
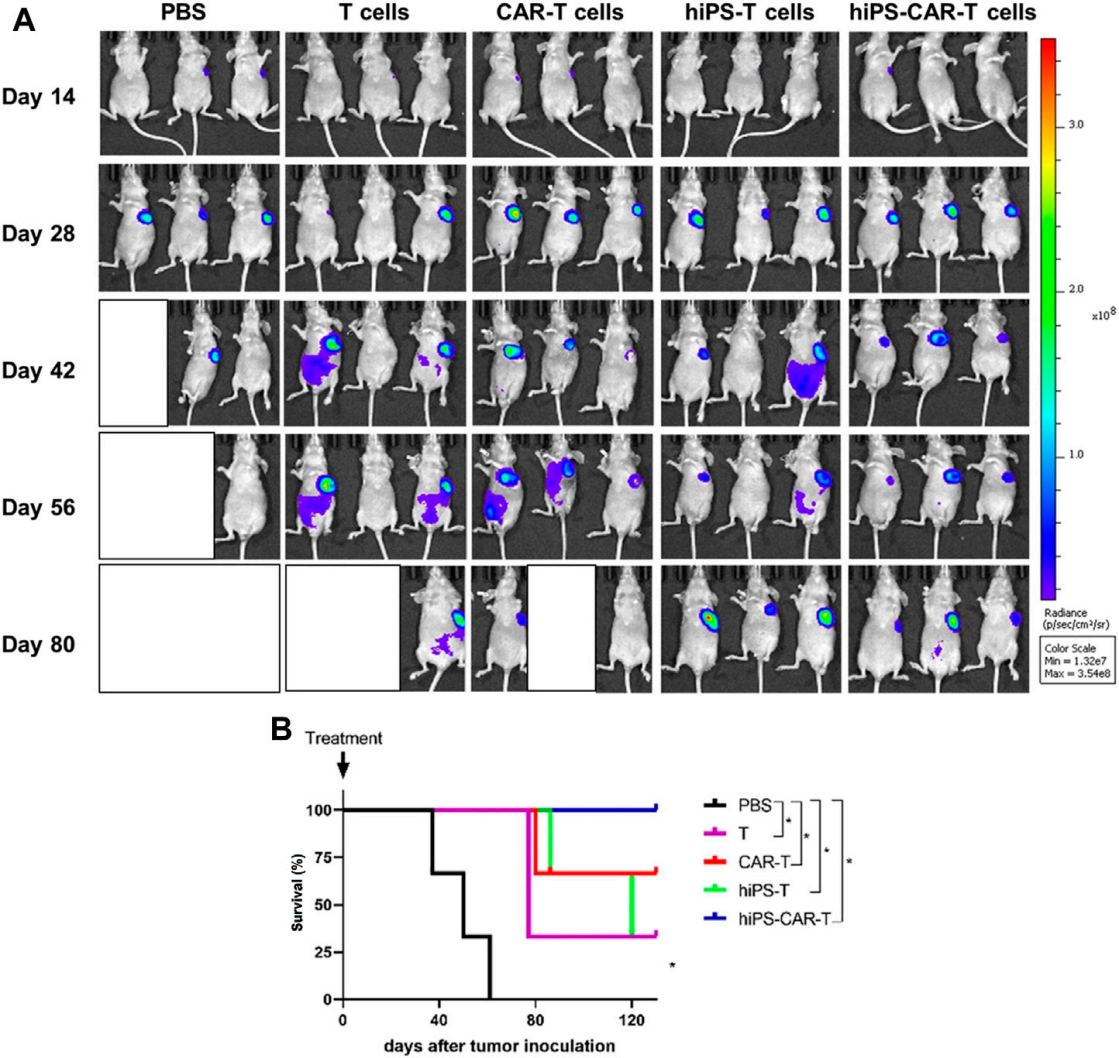
The figure represents co-culture of effector cells and target cells *in vivo*. **(A)** The tumor changes and metastasis were less in the hiPS-CAR-T and CAR-T groups. The hiPSC and T groups gradually developed abdominal metastasis and tumor size after 6 weeks. *In vivo* experiments confirmed that the CAR-T group was more effective in killing and inhibiting tumor growth than the T group, and the function of hiPS-CAR-T cells differentiated from hiPSC was not significantly different from that of peripheral blood (the evaluation results of *in vivo* and *in vitro* experiments were consistent). **(B)** Compared with the PBS negative control group, the life cycle of tumor-bearing mice in the other four groups was prolonged. The tumor-bearing mice in the hiPS-CAR-T group survived for more than 120 days; all tumor-bearing mice in the PBS group died at 61 days *p < 0.05.

In comparison to the PBS negative control group, the other four treatment groups had a much longer life cycle, significantly fewer instances of tumor transformation, and significantly fewer instances of metastasis. By week 6, both the hiPS-T and T groups had a progressive increase in the size of their abdominal metastases (Table 3). Mice who already had tumors and had been treated with hiPS-CAR-T were able to live a normal lifespan without experiencing any signs of the disease, including major tumor growth, metastasis, or mortality. It was shown that CAR-T was superior to the T group in terms of its ability to destroy tumor cells and suppress their proliferation in an animal model. The *in vivo* tumor-suppressing impact of hiPSC differentiation-derived hiPS-T cells and T cells was found to be comparable, according to the results of the live imaging investigations, which suggested that there was no significant difference between the two types of cells. Similar to how there was no significant difference in tumor suppression *in vivo* between hiPS-CAR-T cells prepared from hiPSCs and CAR-T cells prepared from peripheral blood, but the therapeutic effect of immune cells with CAR was better than in the groups of cells without CAR, which is consistent with the results obtained *in vitro* (Figure 5A). In addition, there was a statistically significant difference in the survival curve between each group and the PBS group, with a significance level of *p < 0.05. (Figure 5B).

**TABLE 3:** Continuous tumor size assessment from day 14–80.

	PBS			T cells			CAR-T cells			hiPS-T cells			hiPS-CAR-T cells		
	1	2	3	1	2	3	1	2	3	1	2	3	1	2	3
Day 14	9.97*9.92	9.28*8.28	8.98*8.92	9.28*9.11	9.47*8.99	9.54*8.75	9.97*9.93	9.28*8.33	10.03*9.92	10.17*9.53	9.43*9.37	10.03*9.39	8.4*8.33	9.13*9.06	9.54*9.16
Day 28	10.26*9.69	9.34*8.61	8.91*8.62	10.33*9.12	9.33*8.77	9.61*9.01	10.07*10.01	8.71*8.21	9.77*9.63	10.11*9.11	9.44*9.23	9.79*9.21	8.33*8.21	9.23*9.1	9.33*9.26
Day 42	—	10.07*9.92	8.88*8.53	10.01*8.96	9.21*8.67	9.56*8.97	10.02*9.89	8.98*8.2	10.11*9.93	9.37*8*99	9.23*8.97	9.77*9.3	8.02*8.11	9.16*8.99	8.67*8.66
Day 56	—	—	9.97*8.28	9.89 + 9.01	9.33*7.99	9.53*8.67	9.97*9.96	8.21*8.11	9.69*9.16	9.43*8.39	9.4*8.28	9.28*9.23	8.11*8.1	8.16*8.03	8.56*8.26
Day 80	—	—	—	—	—	9.01*8.79	—	9.01*8.36	8.61*8.13	11.17*10.89	8.72*8.28	9.01*8.88	7.92*7.4	8.03*7.98	8.79*7.79
						(Lon diameter mm*; short diameter mm)									

## 4 Discussion

CAR-T immunotherapy is a cancer treatment method that shows promising results. Herein, we used Raji or A549 cells as target cells for the hiPS-CAR-T. Hu et al. (2020) in their study to develop an anti-CD19 CAR and figure out how well it fights cancer, used Raji cells as their target (Hu et al., 2020). They developed UWC19 and discovered that it successfully destroyed CD19-positive tumor cells while having a low toxicity profile. Some of the studies have used A549 cells as potential targets for CART, including (Wallstabe et al., 2019; Zhang et al., 2019). ROR1-CAR T cells have remarkable antitumor activity in human lung cancer A549 cells. ROR1-CAR T cells infiltrate cancerous tissue and destroy many tumor layers (Hu et al., 2020). EGFRvIII-CART cells accurately and effectively find and eradicate A549-EGFRvIII cells by producing and releasing cytokines such as TNF-α, IFN-γ, granzyme B, and perforin. EGFRvIII-CART cells decreased metastases and improved animal life in mice without causing harm (Zhang et al., 2019).

For iPSCs to be a practical source of readily available anti-tumor T cells, a well-defined and efficient manufacturing technique that can generate sufficient cell numbers for therapeutic usage must be developed. Since then, other groups have been able to *in vitro* generate T lymphocytes from iPSCs using slightly different methods (Themeli et al., 2013). However, by mimicking the identical differentiation pathway used to create human T cells, all of the published approaches successfully recreate the human T cell production process. Following the formation of mesoderm, a definitive hemogenic endothelium is established in induced pluripotent stem cells. Hemogenic endothelium gives rise to a fraction of hematopoietic stem and progenitor cells (HSPC), some of which may eventually differentiate into T cells. Mature CD8 or CD4 SP T lymphocytes, as well as the CD8αβ^+^/CD4^+^ DP cells that give rise to them, are the last, pivotal steps. Given that this is a multi-step differentiation process in which each developmental transition happens with varying efficiency, it stands to reason that the creation of mature SP T cells for therapeutic purposes is challenging. Recently, scientists have been able to generate enough anti-tumor T cells to test their efficacy in xenograft mice models *in vivo* (Themeli et al., 2013). There is, however, no data currently available on how to develop a practical method for synthesizing therapeutically relevant cell numbers. This study developed an efficient and clinically relevant Ff culture method to generate functional CD8^+^ T cells derived from hiPSC. This protocol includes EB formation, hematopoietic induction, differentiation of lymphoid progenitor cells and T cell progenitors, T cell stereotyping, and T cell activation. The protocol we adopted was consistent with that used by the scientists globally for iPSCs differentiation. We proved that hiPSCs generated T cells successfully in all differentiation stages under this feeder-free condition. At the same time, we found that the positive expression rate of CD5 and CD7 could reach more than 95% when 10 ng/mL Interleukin 15 was added during the differentiation into lymphoid progenitor cells (compared with the absence of IL-15, the positive rate of CD5^+^CD7^+^ increased by >70%). After incubation with IL-2, IL-15, and an activator, the DP T cells further matured into CD8^+^ SP T cells. HiPS-CAR-T cells could then be obtained successfully via lentiviral infection. Moreover, we found that they were later able to expand 2-3-fold in normal T cell media.

In most cancers, It is well known that in most cancers, the accumulation of various genetic mutations in benign cells, some responsible for the expression of cancer-specific antigens, is the source of tumorigenesis. With the development of new genetic engineering technology, the rapid generation of “CAR” cells with neoantigen specificity that can target individual patient-specific tumor cells has been widely used in clinics.

Many ongoing clinical trials on treating hematological malignant tumors are showing promising results. However, despite recent advances, there have been reported cases of long-term remission after 2 years or more post-treatment (Maude et al., 2014). The combination of hiPSC and CAR techniques can provide an unlimited supply of tumor-targeting specific T lymphocytes, allowing them to be used more frequently and efficiently in cases. In 2017, the FDA approved axicabtagene ciloleucel, the first CD19-directed CAR T cell therapy drug. In June 2021, this CAR-T drug was approved in China.

Furthermore, in addition to differentiation into CD34^+^ hematopoietic progenitor cells, hiPSCs can further differentiate into other immune cells, including T lineage cells. Due to the dependence on co-cultures of feeder cells, differentiation of hiPSCs into T cells has always been a challenge, leading to high variability caused by the introduction of multiple undefined factors. HiPSCs-derived and CAR-modified hiPS-CAR-T cells show an exceptionally high value when autologous or allogeneic T cells are unavailable. The treatment outcome using adoptive T cells, such as in immunocompromised patients, is unclear. HiPS-CAR-T cells can be used in patients where the isolation of autologous tumor-infiltrating T lymphocytes fails (Goff et al., 2010).

In this study, hiPSCs were induced to differentiate into functional T cells via a serum-free and feeder-free method. Each feeder cell required a different serum and basal medium to maintain culture and co-culture with the hiPSCs, thereby challenging control and reproducibility. Further, feeder-free differentiation systems at the differentiation stage using serum-free media are crucial for developing hiPSC-based T-cell immunotherapies. Therefore, T cells were generated from hiPSCs without a feeder layer in mesoderm formation, hematopoietic specification, lymphoid progenitor differentiation, and T cell progenitor maturation. Genetic modification at the hiPS-T cell level can provide ideal immunotherapeutic properties for the generated immune effectors (hiPS-CAR-T cells).

CAR-T cells, a kind of self-replicating cell, have significantly boosted the effectiveness of cancer therapy (Mehrabadi et al., 2022). One example of how discoveries made in the laboratory may have practical applications in medicine is the creation of CAR T cell therapy. With the advent of cutting-edge CAR-T cell therapies, cancer therapy has gained significant momentum in recent years (Sheykhhasan et al., 2022). Despite its potential to 1 day replaces the vast majority of transplants, CAR T-cell therapy is now only licensed to treat patients who do not respond to transplants or who have post-transplant recurrence (Hamadani et al., 2022). The potential of CAR-T cells as a therapy for cancer depends on how well and how safely they can be produced (Pan et al., 2022). Despite recent successes in some clinical trials, CAR-T cell therapy has resulted in significant morbidity and mortality due to related toxicities (Smith et al., 2022). Because of serious side effects such cytokine release syndrome and neurotoxicity, CAR-T cell therapy is seldom used in clinical practice. Understanding the cause of toxicity in CAR-T cell therapy should lead to major improvements in the treatment’s tolerability. While CAR therapy has been very effective in treating haematological malignancies, the primary goal here is to evaluate its use in the treatment of non-hematological tumors (Lu and Jiang, 2022). To overcome the multiple obstacles that CAR-T cell treatment encounters in solid tumors, novel immunotherapy strategies will likely be necessary. Anti-tumor drugs such as radiation therapy, chemotherapy, immune checkpoint inhibitors, and oncolytic viruses may complement CAR-T cell therapy in the treatment of solid tumors. Nevertheless, given the large diversity of oncolytic viruses, selecting which to utilize in combination with CAR therapy does not seem to be a simple issue. CAR-T therapy success rates vary from cancer type to cancer type. Yet, there is still a lot to learn about the efficacy of this immunotherapy since it is only just being developed (Gill et al., 2022; Rurik et al., 2022). In this study, the serum-free and feeder-free method used is not entirely new method, but it is relatively new in terms of IL-15, which was added as the inducer. During the induction of differentiation, 10 ng/mL of IL-15 was added during the differentiation and preparation of lymphoid progenitor cells and T lineage cells. Further, a lot of experiments found no significant difference in the immune function of T and CAR-T cells from stem cells and peripheral blood.

## 5 Conclusion

Taking together this study revealed that hiPSC CAR-T cells were significantly produced by employing this model. Results revealed anticancer effects of hiPSC CAR-T cells against Ranji or A549 cancer cells thereby inhibiting the proliferation, migration and invasion, and cell cycle. Moreover, the *in vivo* evaluation also showed the inhibitory effects of hiPSC CAR-T treatment. Overall, the combination of hiPSC and CAR provides a new potential source, such as ready-made T cells, with the generation of predicted antigen specificity and cytotoxicity. Given the multifunctional nature of pluripotent stem cells and CAR, this system could facilitate the generation of different T cell subsets through additional genetic modifications and specificity for a range of indications.

## Data Availability

The original contributions presented in the study are included in the article/Supplementary Material, further inquiries can be directed to the corresponding author.
