# Supplementary material for: Regeneration of T cells from human-induced pluripotent stem cells for CAR-T cell medicated immunotherapy
Source: Front Bioeng Biotechnol. 2023 May 18;11:1159507. doi: 10.3389/fbioe.2023.1159507 (PMC10233047; doi:10.3389/fbioe.2023.1159507)
Supplement: Supplementary file 1 [file Table1.DOCX]

Supplementary Material

## Supplementary Figure

**
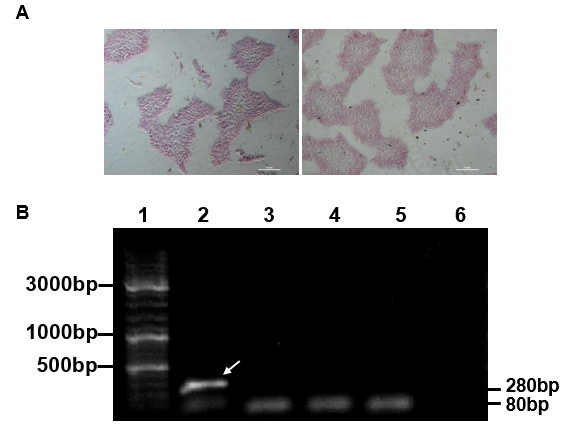
**

**Supplemental Fig S1 Detection of stem cells before hiPSC differentiation.**

(A) The alkaline phosphatase (AP) staining of hiPSC was positive (pink), and hiPSC remained in the state of undifferentiated stem cells.

(B) Mycoplasma assay was performed on hiPSC and CD19+ Raji-luc+ cells, respectively. The results showed that both cells were not infected with Mycoplasma and were available for subsequent experiments.

1: DNA Ladder (0.1-10 KB)

2: Mycoplasma male ginseng

3. Mycoplasma female ginseng

4: Supernatant samples of cells to be detected (hiPSC)

5: Supernatant samples of cells to be detected (CD19^+^ Raji-Luc+)

6. Double steaming water without mycoplasma
